# Sex and Age Differences in Outcomes of Traumatic Brain Injury: Findings from the Japan Neurotrauma Data Bank

**DOI:** 10.3390/jcm15052034

**Published:** 2026-03-06

**Authors:** Yasuhiro Nakajima, Takahiko Yoshimoto, Mariko Kurihara, Akihito Kato, Jun Sasaki, Akatsuki Kokaze, Kenji Dohi

**Affiliations:** 1 Department of Emergency and Disaster Medicine, Showa Medical University School of Medicine, Tokyo 142-8555, Japan; runners.md@med.showa-u.ac.jp (Y.N.); mariko-kurihara@med.showa-u.ac.jp (M.K.); akihito@med.showa-u.ac.jp (A.K.); jun-sa@da2.so-net.ne.jp (J.S.); 2 Department of Hygiene, Public Health and Preventive Medicine, Showa Medical University School of Medicine, Tokyo 142-8666, Japan; yoshimotot@med.showa-u.ac.jp (T.Y.); akokaze@med.showa-u.ac.jp (A.K.); 3 Japan Neurotrauma Data Bank (JNTDB) Committee (Japan Society of Neurotraumatology), Tokyo 162-0052, Japan

**Keywords:** traumatic brain injury, sex differences, prognosis, aging, Japan Neurotrauma Data Bank

## Abstract

**Background/Objectives:** Traumatic brain injury (TBI) remains a major global health concern, contributing substantially to mortality and long-term disability. Although sex hormones have been proposed to influence TBI outcomes, sex has not been incorporated into widely used prognostic models. Given the rapidly aging population in Japan, this study aimed to investigate the impact of sex on post-TBI outcomes. **Methods**: We analyzed data from the Japan Neurotrauma Data Bank, comprising four prospective multicenter cohorts (P1998, P2004, P2009, P2015). Patients with Glasgow Coma Scale (GCS) scores ≥9 at admission were included. Multivariate logistic regression identified predictors of unfavorable outcomes (death, vegetative state, or severe disability) on the Glasgow Outcome Scale. Subgroup analyses stratified by sex and age were performed. **Results**: Of 717 eligible patients, 195 (27.2%) were females. Females were significantly older than males (median age: 68 vs. 58.5 years). Traffic accidents were more common among females, whereas non-traffic injuries predominated in males. Independent predictors of unfavorable outcomes included age ≥51 years, male sex, GCS 9–12, Injury Severity Score ≥ 16, hypoxia, targeted temperature management, and subarachnoid hemorrhage. Stratified analysis showed that females aged ≥75 years had significantly better outcomes. **Conclusions**: Female sex was independently associated with more favorable functional outcomes among patients with TBI presenting with admission GCS ≥ 9, particularly among those aged ≥75 years. Although prior studies have reported potential biological influences, the underlying mechanisms remain uncertain. Further investigation of sex differences and associated risk factors may help inform the development of more individualized management strategies for patients with TBI.

## 1. Introduction

Traumatic brain injury (TBI) remains a major global health concern, contributing substantially to mortality and long-term disability, and imposing considerable burdens on individuals, families, and healthcare systems [1]. Traditionally, TBI has been a major cause of death among younger individuals. However, in recent decades, its epidemiology has shifted, with incidence rates rising among older adults in developed countries [2], including Japan [3].

As the fastest-aging society worldwide, Japan has experienced a marked rise in its older adult population. In 2022, nearly 30% of the population was aged ≥65 years, projected to reach 35% by 2040 [4]. Yokobori et al. reported that approximately half of all TBIs now occur in patients aged ≥65 years, with a growing number of cases among those aged ≥75 years [3]. Another study on geriatric patients with TBI in Japan, conducted every three years from 2004 to 2018, found a decline in cases among those aged 65–74, while the proportion of patients aged ≥85 years increased from 7.7% in 2004–2006 to 17.8% in 2016–2018 [5].

Regarding sex differences, the proportion of females in Japan increases with age because of their longer life expectancy. The females-to-males ratio is 1.3:1 among those aged ≥65 years, 1.53:1 among those aged ≥75, and 2.11:1 among those aged ≥85 [4]. Several studies [3,5,6,7,8,9,10] have demonstrated that the median age of females with TBI is higher than that of males. However, widely used predictive models for TBI outcomes, such as IMPACT, CRASH, and TRISS, do not include sex as a prognostic factor [11,12,13]. This omission may stem from epidemiological data showing that TBI incidence is significantly higher in males across all age groups [14,15], or from the ongoing debate about the impact of sex on TBI outcomes [16,17].

Furthermore, preclinical studies have highlighted sex differences in TBI for decades, particularly emphasizing the neuroprotective effects of estrogen and progesterone, which have been shown to suppress apoptosis, inhibit oxidative stress, and modulate proinflammatory pathways [18,19]. Therefore, we focused not only on sex differences but also on their relationship across age groups, considering potential associations with changes in female hormone secretion.

Given the growing number of older adults with TBI, particularly females, in the context of an aging society, investigating the role of sex in TBI outcomes is crucial [6,7]. Furthermore, age-related brain characteristics, such as cerebral atrophy leading to enlarged subdural space, might affect initial consciousness levels [20,21]. Consequently, older adults initially diagnosed with mild TBI may worsen to severe TBI. Preventing secondary brain injury is therefore essential in preventing clinical worsening [22,23]. The data bank used in this study included participants with Glasgow Coma Scale (GCS) scores of ≤8 on hospital admission, those who deteriorated within 48 h despite having GCS scores of ≥9 upon admission, or undergoing neurosurgical interventions, despite an initial Glasgow Coma Scale (GCS) score of ≥9 upon admission. In the JNTDB, patients with an initial Glasgow Coma Scale (GCS) score of ≥9 generally correspond to a characteristic clinical pattern of traumatic brain injury in older adults, in which patients are initially diagnosed as having mild to moderate TBI at admission but subsequently deteriorate after hospital admission [20,21]. In traumatic brain injury, primary brain injury resulting from the initial mechanical impact often involves structural damage that is largely irreversible [24]. In patients with severe traumatic brain injury (GCS ≤ 8), outcomes are strongly influenced by the severity of the initial injury, and high mortality rates, as well as a high proportion of unfavorable outcomes, have been reported [25]. By contrast, patients presenting with an admission GCS ≥ 9 represent a population in which functional outcomes may not be determined solely by primary injury but may instead reflect subsequent clinical progression. By focusing on this subgroup, we aimed to evaluate sex- and age-related differences in a population in which outcomes may have been influenced by biological or systemic modifying factors.

## 2. Materials and Methods

### 2.1. Study Design and Participants

This study utilized data from the Japan Neurotrauma Data Bank (JNTDB), which was established by the Japan Society of Neurotraumatology in 1998 [2]. The JNTDB comprises data from intermittent 2-year studies. Four separate projects, P1998, P2004, P2009, and P2015, were conducted, involving 10, 19, 22, and 33 facilities, respectively. The JNTDB primarily focuses on patients with severe TBI. The inclusion criteria for severe TBI were as follows: (1) GCS score of ≤8 upon hospital admission or within 48 h of admission. (2) Deterioration to GCS ≤ 8 within 48 h despite an initial GCS ≥ 9 upon admission; or (3) Neurosurgical intervention, including placement of intracranial pressure (ICP) monitoring, despite an initial GCS ≥ 9. (4) All ages were included, except in P1998, which excluded patients aged <6 years. A total of 4539 patients were registered across the four projects. For this study, we used data from all four projects, including sex, age, project, length of hospital stay, cause of trauma (traffic accidents, non-traffic accidents, and others), systolic blood pressure (SBP), heart rate, body temperature (BT), GCS, pupil dilatation, blood sugar level, partial pressure of carbon dioxide (PCO_2_), partial pressure of oxygen (PO_2_), blood pH, Injury Severity Score (ISS), injury site, head Abbreviated Injury Scale (AIS), TBI type (I-V, evacuated mass, or non-evacuated mass), skull fracture (vault or basal), intracranial air, intracranial foreign body, intraventricular hemorrhage (IVH), subarachnoid hemorrhage (SAH), surgical intervention, targeted temperature management (TTM), and Glasgow Outcome Scale (GOS) score.

**Figure 1:**
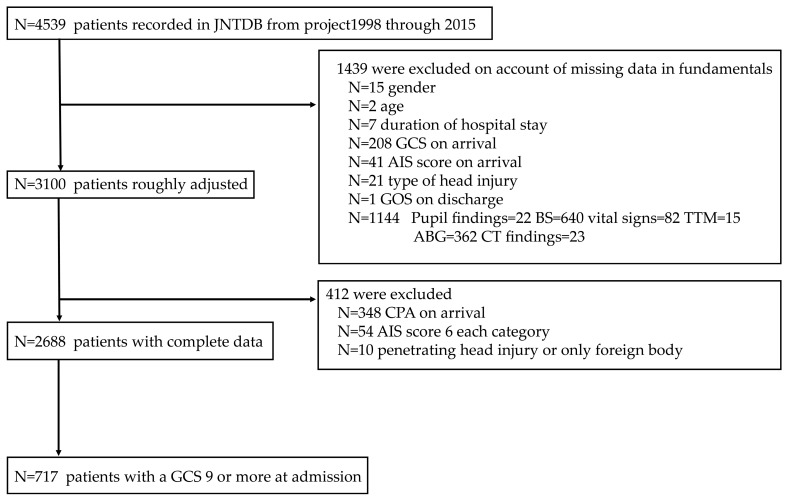
Flow diagram of patient selection and inclusion in the study. Flow diagram illustrating the selection process of patients from the Japan Neurotrauma Data Bank (P1998–P2015). Patients with an admission Glasgow Coma Scale (GCS) score ≥9 were included in the present analysis. Exclusion criteria and final sample size are shown. A total of 2688 patients were included from four projects (P1998, P2004, P2009, and P2015). JNTDB, Japan Neurotrauma Data Bank; GCS, Glasgow Coma Scale; AIS, Abbreviated Injury Scale; GOS, Glasgow Outcome Scale; CPA, Cardiopulmonary arrest; BP, blood pressure; HR, Heart rate; BS, Blood sugar; TTM, Targeted temperature management; SAH, Subarachnoid hemorrhage; IVH, Intraventricular hemorrhage.

A total of 1439 patients were excluded because of missing data on key analytical variables. In addition, 412 patients were excluded because of cardiopulmonary arrest upon arrival, AIS scores > 6 in any category, or ISS of 75. Only patients with GCS scores ≥9 upon admission were included. Ultimately, 717 patients met the inclusion criteria (Figure 1).

### 2.2. Study Endpoints

The primary outcome was assessed using the GOS, a 5-point scale (death, vegetative state, severe disability, moderate disability, and good recovery) used to assess the functional outcome of patients following TBI. This study categorized GOS scores into unfavorable (death, vegetative state, or severe disability) and favorable (moderate disability or good recovery) outcomes. The primary endpoint was the occurrence of an unfavorable outcome at hospital discharge. To address potential bias related to discharge-based outcome assessment, 6-month GOS was additionally evaluated as a secondary outcome to assess the robustness of the primary findings.

### 2.3. Statistical Analysis

Baseline characteristics were compared between males and females using the chi-square test or Fisher’s exact test for qualitative variables and the Wilcoxon rank-sum test for quantitative variables. To evaluate factors associated with unfavorable outcomes, a logistic regression model was used to calculate odds ratios (ORs) and 95% confidence intervals (CIs). Factors included in the analysis were selected based on clinical fundamentals and previous research [1,3,5,6,7]. The factors included age group (<15, 16–50, 51–64, 65–74, and ≥75 years), sex, length of hospital stay, cause of trauma (traffic or non-traffic accident), GCS on admission, pupillary findings (unilateral or bilateral dilation), low blood pressure (systolic BP < 90 mmHg), bradycardia (heart rate < 60 beats per minute), hyperthermia (BT ≥ 37.5 °C), ISS ≥ 16, hyperglycemia (BS ≥ 200 mg/dL), hypercapnia (PCO_2_ > 45 mmHg) or hypocapnia (PCO_2_ < 35 mmHg), hypoxia (PO_2_ < 60 mmHg), acidemia (pH < 7.3), TCDB classification, operation for TBI, TTM, skull fracture, SAH, and IVH.

The same logistic regression model was further used to evaluate the association between favorable outcomes and sex stratified by age group. In the secondary analysis, multivariable logistic regression was repeated using 6-month unfavorable outcomes as the dependent variable. Paired comparisons between discharge and 6-month outcomes were performed using the Wilcoxon signed-rank test. Changes in the marginal distribution of outcomes were assessed using Bowker’s test of symmetry. To examine whether changes in functional status between discharge and 6 months differed by sex, transitions in GOS were categorized as improved, unchanged, or worsened. Comparisons between males and females were performed using the chi-square test. This analysis was conducted for both the 5-point GOS and dichotomized outcomes.

A two-sided *p*-value < 0.05 was considered statistically significant. JMP Pro 17 (SAS Institute Inc., Cary, NC, USA) was used for all statistical analyses.

## 3. Results

**Figure 2:**
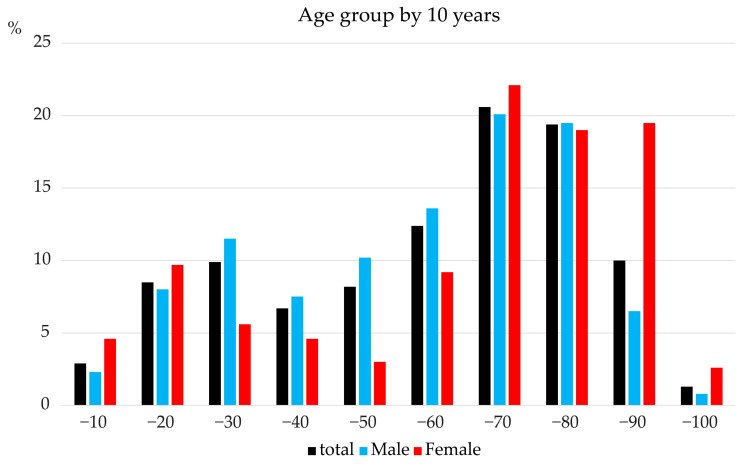
Age distribution of patients with traumatic brain injury (GCS ≥ 9) stratified by sex. Bar graph showing the age distribution of male and female patients with traumatic brain injury and an admission GCS score ≥9, categorized in 10-year age intervals.

Based on the inclusion and exclusion criteria, 717 patients were included in the study (Table 1). Of these, 195 (27.2%) were females, with a median age of 68 years (interquartile range [IQR]: 41–79). Females were significantly older than males (*p* < 0.001). The largest age group for females was those aged ≥75 years (33.9%), while that for males was the 16–50 age group (35.6%). Figure 2 illustrates the age distribution between males and females across 10-year periods, showing a small peak in the teens and twenties and a larger peak in the fifties to seventies. Patients aged ≥75 years.

The baseline demographic and clinical characteristics are summarized in Table 1. The median length of hospital stay was 27 days (IQR: 13–50), with no significant differences between males (median: 27 days, IQR: 14–49) and females (median: 28 days, IQR: 12–52) (*p* = 0.702). Traffic accidents were more frequent among females (53.3%) than among males (37%) (*p* < 0.001). For females, 44.2% of traffic accidents involved pedestrians, whereas for males, bicycles were the most common cause (33.2%). Falls on the ground level or from stairs were the most frequent cause of non-traffic accidents for both sexes (males, 71.6%; females, 81.3%).

Regarding vital signs, females were more likely to present with SBP < 90 mmHg (*p* = 0.041); however, no significant differences were observed in temperature or bradycardia (*p* = 0.342 and *p* = 0.254, respectively). GCS scores on admission did not differ significantly between males and females (*p* = 0.238). Similarly, no significant differences were observed in pupil dilatation (*p* = 0.636) or arterial blood gas values, including acidemia, hyperglycemia, hypo- and hypercapnia, and hypoxia (*p* = 0.467, *p* = 0.454, *p* = 0.123, *p* = 0.181, and *p* = 0.881, respectively).

85.2% of patients had ISS ≥ 16, with no significant sex differences (*p* = 0.076). Head AIS scores of 5 were the most frequent severity type for both sexes (males, 45%; females, 40%), with no significant differences in the overall distribution of injury severity (*p* = 0.702). For TCDB classification, Type V (evacuated mass) was the most common in both groups, with no significant differences (*p* = 0.758).

Computed tomography findings revealed that vault fractures were more common in males (*p* = 0.0049); however, no significant differences were found for skull fractures, basal fractures, traumatic pneumocephalus, intracranial foreign body, IVH, or SAH (*p* = 0.780, *p* = 0.287, *p* = 0.232, *p* = 0.493, and *p* = 0.798, respectively). Furthermore, no significant differences were observed in the rates of operation or TTM between males and females (*p* = 0.909 and *p* = 0.424, respectively).

GOS scores at hospital discharge showed that good recovery was the most frequent outcome, occurring in 30.8% of patients, with no significant differences between males (30.5%) and females (31.8%) (*p* = 0.910).

**Figure 3:**
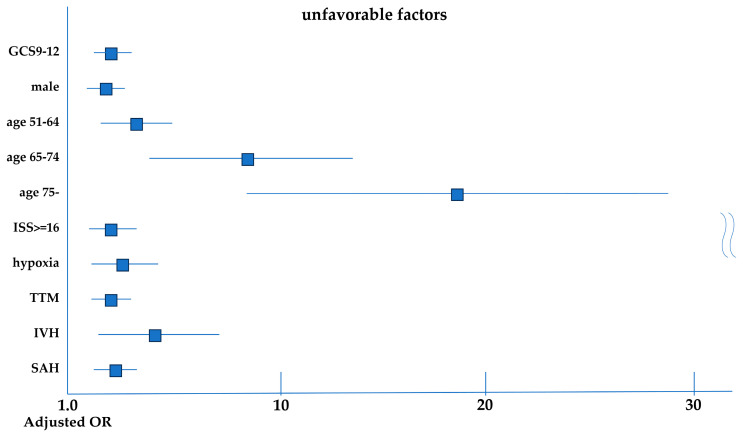
Multivariable logistic regression analysis of factors associated with unfavorable outcomes at discharge. Forest plot showing adjusted odds ratios (ORs) with 95% confidence intervals (CIs) derived from multivariable logistic regression analysis for factors associated with unfavorable functional outcomes at hospital discharge. Male sex was used as the reference category.

Table 2 identifies several risk factors associated with unfavorable outcomes. In the multivariable logistic regression analysis, significant risk factors for unfavorable outcomes included ages 51–64, 65–74, and ≥75 years (adjusted OR: 2.79, 95% CI: 1.65–4.74; adjusted OR: 6.09, 95% CI: 3.58–10.36; and adjusted OR: 15.67, 95% CI: 8.51–28.84, respectively) compared with the 16–50 years age group. Additional significant risk factors were male sex (adjusted OR: 1.78, 95% CI: 1.14–2.78), GCS scores of 9–12 on admission (adjusted OR: 2.18, 95% CI: 1.48–3.21), ISS ≥ 16 (adjusted OR: 1.76, 95% CI: 1.01–3.06), hypoxia (adjusted OR: 2.00, 95% CI: 1.02–3.90), TTM (adjusted OR: 1.95, 95% CI: 1.27–2.98), IVH (adjusted OR: 2.57, 95% CI: 1.06–6.28) and SAH (adjusted OR: 2.19, 95% CI: 1.45–3.31) (Figure 3).

**Figure 4:**
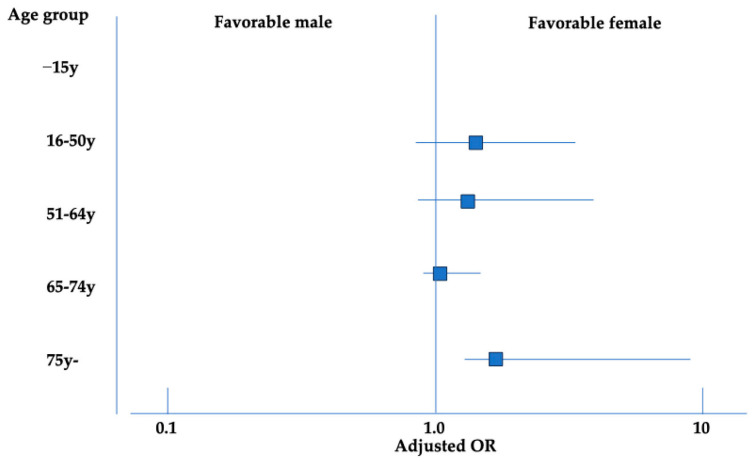
Age-stratified analysis of the association between sex and unfavorable outcomes at discharge. Forest plot showing adjusted odds ratios (ORs) with 95% confidence intervals (CIs) for females (reference: males), calculated using multivariable logistic regression models within each age group.

Table 3 shows the age distribution of outcomes stratified by sex. Among females, 45.6% experienced unfavorable outcomes, with a median age of 76 (68–82.5) years in the unfavorable group, significantly higher than the median age of 53 (20.8–69.3) years in the favorable group (*p* < 0.001). Patients aged ≥75 years were more common in the unfavorable group, whereas those aged 16–50 predominated in the favorable group. Among males, 47.9% had unfavorable outcomes, with a median age of 68 (56.8–78.0) years compared with 43.5 (25.0–62.0) years in the favorable group (*p* < 0.001). Similarly, the unfavorable group had a higher proportion of patients aged ≥75 years, while the favorable group had more patients aged 16–50. Figure 4 illustrates the sex differences across age groups based on multivariable logistic regression analysis using the same factors as in Table 2. Females tended to have more favorable outcomes than males in the 16–50, 51–64, and 65–74 age groups with no significant differences (adjusted OR: 2.25, 95% CI: 0.75–6.74; adjusted OR: 2.2, 95% CI: 0.66–7.25; and adjusted OR: 1.18, 95% CI: 0.66–2.1, respectively). However, among those aged ≥75 years, females showed significantly more favorable outcomes than males (adjusted OR: 3.2, 95% CI: 1.08–9.54). Six-month GOS data were available for 521 patients. Multivariable analysis using 6-month unfavorable outcome as the dependent variable yielded findings consistent with the primary discharge-based analysis (Supplementary Table S1). Paired analyses demonstrated a significant improvement in GOS at 6 months compared with discharge, as confirmed by both the Wilcoxon signed-rank test and Bowker’s test of symmetry (both *p* < 0.001). The corresponding 5-point transition matrix is presented in Supplementary Table S2. Similar results were obtained when outcomes were dichotomized (both the Wilcoxon signed-rank test and Bowker’s test of symmetry, both *p* < 0.001), with the detailed 2 × 2 transition table provided in Supplementary Table S3. Changes in GOS between discharge and 6 months did not differ significantly between males and females. Using the 5-point scale, improvement was observed in 18.8% of males and 16.8% of females, worsening in 3.7% and 4.9%, and no change in 77.5% and 78.3%, respectively (*p* = 0.74) (Supplementary Table S4). When outcomes were dichotomized, improvement occurred in 7.1% of males and 8.4% of females, worsening in 1.1% and 2.1%, and no change in 91.8% and 89.5%, respectively (*p* = 0.57) (Supplementary Table S5).

## 4. Discussion

The analysis included 717 patients, categorized as having moderate and mild TBI on admission. Logistic regression analysis identified male sex as a risk factor for poor prognosis in patients with TBI. Other associated factors included older age, GCS scores of 9–12 upon admission, ISS ≥ 16, hypoxia, TTM, IVH, and SAH. Notably, when 6-month unfavorable outcome was used as the dependent variable, multivariable analysis yielded similar trends, with male sex remaining associated with poorer outcomes. These findings support the robustness of the primary discharge-based analysis.

Regarding sex differences in TBI, numerous studies have reported controversial findings [6,7,8,10]. Gupte et al. reported that 47% of 156 human studies showed worse outcomes in females than in males, and 26% demonstrated better outcomes in females [26]. However, outcomes varied depending on the study population and TBI severity. After stratifying studies by severity (non-stratified, mild-moderate, and moderate-severe), 33% (N = 48), 9% (N = 67), and 46% (N = 41) of studies, respectively, showed better outcomes in females than in males. Furthermore, after classifying the studies by sample size—small (0–1000), medium (1000–10,000), and large (>10,000)—23% (N = 118), 17% (N = 23), and 67% (N = 15) of studies, respectively, showed better outcomes in females than in males [26]. Although our study falls into the small-sample category, it demonstrated better outcomes in females than in males. These differences might have been influenced by the sample size classification and the inclusion of all TBI severities [26]. Conversely, a previous study focusing on patients with head AIS scores of 3–5 reported better outcomes in females than in males [7].

Shahrokhi et al. have demonstrated the neuroprotective role of estrogen and progesterone in improving ICP, cerebral perfusion pressure, and neurological scores following TBI in ovariectomized rats [27]. Similarly, Nasre et al. reported that progesterone reduced brain edema and lesion volume in a systematic review and meta-analysis of 48 preclinical animal studies [28]. Roof et al. demonstrated that the exogenous administration of both estrogen and progesterone improved outcomes after cerebral ischemia and TBI in experimental models [29]. Zhou et al. suggested that progesterone reduces the activation and infiltration of inflammatory cells, including microglia and neutrophils, in injured brain tissue by regulating immune and inflammatory responses through regulatory T cell or IL-10 pathways [30]. Likewise, Guennoun reported that findings from the reviewed literature indicate that progesterone possesses substantial neuroprotective potential and may play a therapeutic role in attenuating brain damage [31].

Considering the effects of female hormones on TBI, some studies have focused on the relationships between sex and age, especially menopause [32,33]. In the present study, 50 years was used as a surrogate cutoff for menopausal status, based on a large systematic review and meta-analysis of 46 population-based studies across six countries [34]. The present study investigated not only sex differences but also their relationships across age groups. Females tended to have more favorable outcomes than males in the 16–50, 51–64, and 65–74year age groups; however, these differences were not significant. Conversely, the ≥75-year age group showed significant differences, partially consistent with findings from a previous study in Japan [7]. The tendency was less pronounced in the 65–74-year age group than in the 16–50 and 51–64-year age groups, which may reflect age-related changes in hormonal status. Estradiol levels decline substantially during menopause and continue to decrease progressively with age [35,36]. However, the significant differences observed in the ≥75-year age group may not be fully explained by estrogen alone. Davis et al. evaluated outcomes in 13,000 patients with TBI stratified by age (premenopausal and postmenopausal) and found improved outcomes in postmenopausal patients [32]. They observed that females had significantly lower mortality and morbidity than males with peri- and post-menopausal females exhibiting the lowest mortality after adjustment for risk factors [37]. On this basis, they concluded that estrogen did not appear to provide a neuroprotective benefit in patients with moderate-to-severe TBI [37]. Consistently, Davis et al. also reported that the endogenous production of female sex hormones was not associated with neuroprotection [32]. As sex hormone levels decline after menopause, these differences are unlikely to be explained by estrogen alone. Arnold et al. suggested that hormonal influences alone do not fully account for sex-related differences in the brain and discussed the potential role of sex chromosome composition [38]. In addition to reported associations between sex and cerebral blood flow as well as genetic factors related to neuroprotection, the disparity in life expectancy between older males and females, particularly in Japan, where life expectancy is 81.09 years for males and 87.14 years for females [4,39], may reflect broader biological and functional differences between sexes. Similarly, Guo et al. reported that sarcopenia and frailty significantly delay functional recovery and are independent predictors of adverse surgical outcomes in geriatric patients [40]. Within the context of TBI, Zacchetti L et al. found that higher scores on the Clinical Frailty Scale were significantly associated with poor neurological outcomes 6 months post-injury [41]. These findings raise the possibility that differences in life expectancy may be related to outcome variability in older populations. Hosomi et al. reported that, across 10-year age categories, male patients consistently showed higher mortality rates than age-matched females, particularly among those aged 60–69, 70–79, 80–89, and 90–99 years. They suggested that differences in overall physical condition might partly explain these sex-based disparities in outcome [7]. Physical conditions such as congestive heart failure, diabetes mellitus, hypertension, chronic kidney disease, liver disease, and medications (especially antiplatelet drugs and anticoagulants), which this present study cannot exclude, might affect outcomes as confounders [7,10,11,42]. We focused on TBI cases with GCS scores ≥9 on admission. In older adults, cerebral atrophy and the resulting enlargement of cerebrospinal fluid spaces may provide compensatory buffering against new intracranial lesions. Consequently, increases in ICP are less frequent, and the GCS can underestimate the actual severity of brain injury [43]. In an aging society, the phenomenon of “talk and die,” characteristic of older adults with TBI, will probably increase. Therefore, the risk factors for this phenomenon warrant further investigation. There were some limitations to our study. First, we analyzed data from four projects from P1998 to P2015, which did not account for temporal changes in the management of TBI, including the implementation of neurocritical care [2,44,45]. Second, we used complete data with included variables in our analysis to integrate all projects, which may not fully account for the characteristics of excluded data. Third, we did not consider certain physical conditions, such as congestive heart failure (CHF), diabetes mellitus (DM), hypertension (HT), chronic kidney disease (CKD), liver disease, and medications. Adding these factors may provide further insight into the sex differences in TBI. Fourth, in the present study, GOS at hospital discharge was used as the primary endpoint. However, a previous study has indicated that patients discharged home showed functional improvement compared with those transferred to other institutions [46], suggesting that discharge destination and post-acute management strategies may influence subsequent functional outcomes. The JNTDB is primarily an acute-phase registry of traumatic brain injury. Although a 6-month GOS is recorded, information regarding discharge destination is limited to broad categories, such as discharge home or transfer to another hospital or facility. In Japan, discharge destinations vary widely, including long-term care institutions that do not necessarily provide structured rehabilitation. In the present study, among patients with available 6-month follow-up data, significant improvement in GOS was observed in both males and females compared with discharge. However, due to the structure of the registry, it was not possible to identify the specific factors contributing to this improvement. Future investigations that include long-term follow-up and detailed information on post-discharge management and rehabilitation may help to clarify these issues. Finally, although prior experimental and clinical studies have suggested potential hormonal and genetic mechanisms underlying sex differences in TBI outcomes, the present study did not include direct measurements of sex hormone levels, menopausal status, or hormone replacement therapy. Therefore, the biological interpretations discussed herein should be regarded as exploratory and warrant further investigation in studies incorporating endocrine and molecular data.

## 5. Conclusions

Female sex was independently associated with more favorable outcomes among patients with TBI presenting with admission GCS ≥ 9. Notably, the most pronounced differences were observed in the ≥75-year age group, although the underlying mechanisms remain uncertain. The present registry-based cohort study did not include direct measurements of sex hormone levels, sex chromosome composition, frailty-related factors, or comorbid physical conditions. Further investigation of sex differences and associated risk factors may help inform the development of more individualized management strategies for patients with TBI.

## Figures and Tables

**Table 1:** Baseline demographic and clinical characteristics of patients with traumatic brain injury (GCS ≥ 9) stratified by sex.

		Total (N = 717)	Male (N = 521, 72.7%)	Female (N = 195, 27.2%)	*p*-Value
Age	median, (IQR)	62 (36–74)	58.5 (35–71)	68 (41–79)	<0.001
**age group**					<0.001
0–15	n, (%)	34 (4.7)	20 (3.8)	14 (7.2)	
16–50	n, (%)	226 (31.5)	186 (35.6)	40 (20.5)	
51–64	n, (%)	140 (19.5)	111 (21.3)	29 (14.9)	
65–74	n, (%)	159 (22.2)	113 (21.7)	46 (23.6)	
≥75	n, (%)	158 (22)	92 (17.6)	66 (33.9)	
					
**length of hospital stay**	median, (IQR)	27 (13–50)	27 (14–49)	28 (12–52)	0.702
**Cause of trauma**					<0.001
traffic accident	n, (%)	297 (41.4)	193 (37.0)	104 (53.3)	
non-traffic accident	n, (%)	415 (57.9)	324 (62.1)	91 (46.7)	
Unknown	n, (%)	5 (0.7)	5 (1.0)	0 (0.0)	
**Causes of traffic accident (N = 297)**					
motor vehicle driver	n, (%)	19 (2.6)	18 (9.3)	1 (1.0)	
motor vehicle passenger	n, (%)	3 (0.4)	1 (0.5)	2 (1.9)	
back seat passenger	n, (%)	7 (1.0)	3 (1.6)	4 (3.9)	
unknown	n, (%)	2 (0.3)	2 (1.0)	0 (0.0)	
motorcycle driver	n, (%)	44 (6.1)	37 (19.2)	7 (6.7)	
motorcycle passenger	n, (%)	0 (0.0)	0 (0.0)	0 (0.0)	
Unknown	n, (%)	31 (4.3)	24 (12.4)	7 (6.7)	
Bicycle	n, (%)	101 (14.1)	64 (33.2)	37 (35.6)	
Pedestrian	n, (%)	90 (12.6)	44 (22.8)	46 (44.2)	
**Causes of non-traffic accident (N = 415)**					
fall from height	n, (%)	51 (12.3)	44 (13.6)	7 (7.7)	
fall on the ground level or from stairs	n, (%)	306 (73.7)	232 (71.6)	74 (81.3)	
Bruise	n, (%)	19 (4.6)	17 (5.2)	2 (2.2)	
assault/abuse	n, (%)	1 (0.2)	1 (0.3)	0 (0.0)	
Others	n, (%)	1 (0.2)	1 (0.3)	0 (0.0)	
Unknown	n, (%)	37 (8.9)	29 (8.6)	8 (8.8)	
**Clinical data**					
systolic BP < 90 mmHg	n, (%)	32 (4.5)	18 (3.5)	14 (7.2)	0.041
HR < 60 beats per minute	n, (%)	37 (5.2)	30 (5.8)	7 (3.6)	0.342
BT ≥ 37.5 °C	n, (%)	51 (7.1)	41 (7.9)	10 (5.1)	0.254
**Glasgow Coma Scale (GCS)**					0.238
9–12	n, (%)	321 (44.8)	241 (46.2)	80 (41.0)	
13–15	n, (%)	396 (55.2)	281 (53.8)	115 (58.9)	
					
**Dilatation of pupils**					0.636
Bilateral	n, (%)	17 (2.4)	13 (2.5)	4 (2.1)	
Unilateral	n, (%)	40 (5.6)	31 (5.9)	9 (4.6)	
					
**Blood test**					
BS ≥ 200 mg/dL	n, (%)	135 (18.8)	102 (19.5)	33 (16.9)	0.454
PCO_2_ < 35 mmHg	n, (%)	214 (29.8)	162 (31.0)	52 (26.7)	0.123
PCO_2_ > 45 mmHg	n, (%)	98 (13.7)	77 (14.8)	21 (10.8)	0.181
PO_2_ < 60 mmHg	n, (%)	61 (8.5)	44 (8.4)	17 (8.7)	0.881
acidemia pH < 7.3	n, (%)	22 (3.1)	18 (3.5)	4 (2.1)	0.467
**Severity and Classification of trauma**					
ISS ≥ 16	n, (%)	611 (85.2)	437 (83.7)	174 (89.2)	0.076
					
head AIS					
3	n, (%)	92 (12.8)	68 (13.0)	24 (12.3)	
4	n, (%)	264 (36.8)	184 (35.3)	80 (41.0)	
5	n, (%)	313 (43.7)	235 (45.0)	78 (40.0)	
					
**TCDB classification (classification of TBI)**					0.758
I	n, (%)	19 (2.7)	12 (2.3)	7 (3.6)	
II	n, (%)	110 (15.3)	79 (15.1)	31 (15.9)	
III	n, (%)	17 (2.4)	13 (2.5)	4 (2.1)	
IV	n, (%)	5 (0.7)	4 (0.8)	1 (0.5)	
V (evacuated)	n, (%)	426 (49.4)	317 (60.1)	109 (55.9)	
VI (non–evacuated)	n, (%)	140 (19.5)	97 (18.6)	43 (22.1)	
					
**total skull fracture**	n, (%)	515 (71.8)	373 (71.5)	142 (72.8)	0.780
**vault skull fracture**	n, (%)	426 (59.4)	327 (62.6)	99 (50.1)	0.005
**basal skull fracture**	n, (%)	173 (24.1)	124 (23.8)	49 (25.1)	0.153
**traumatic pneumocephalus**	n, (%)	131 (18.3)	89 (17.1)	42 (21.5)	0.287
**intracranial foreign body**	n, (%)	18 (2.5)	16 (3.1)	2 (1.0)	0.232
**intraventricular hemorrhage (IVH)**	n, (%)	45 (6.3)	35 (6.7)	10 (5.1)	0.493
**subarachnoid hemorrhage (SAH)**	n, (%)	424 (59.1)	307 (58.8)	117 (60.0)	0.798
					
**Operation**	n, (%)	595 (83.8)	434 (84.0)	161 (83.4)	0.909
**targeted temperature management (TTM)**	n, (%)	240 (33.5)	170 (32.6)	70 (35.9)	0.424
					
**Outcome at discharge (GOS: Glasgow Outcome Scale)**					0.910
D (dead)	n, (%)	128 (17.9)	94 (18.0)	34 (17.4)	
VS (vegetative state)	n, (%)	47 (6.6)	37 (7.1)	10 (5.1)	
SD (severe disability)	n, (%)	164 (22.9)	119 (22.8)	45 (23.1)	
MD (moderate disability)	n, (%)	157 (21.9)	113 (21.7)	44 (22.6)	
GR (good recovery)	n, (%)	221 (30.8)	159 (30.5)	62 (31.8)	
					
**Discharge destination**					0.853
discharged to home	n, (%)	254 (35.4)	185 (35.4)	69 (35.4)	
discharged to another hospital/a facility	n, (%)	225 (31.4)	159 (30.5)	66 (33.8)	
Unknown	n, (%)	13 (1.8)	9 (1.7)	4 (2.1)	

**Table 2:** Multivariable logistic regression analysis of factors associated with unfavorable outcomes at hospital discharge.

	Adjusted OR	95%CI	Crude OR	95%CI
**length of hospital stay**				
<30	reference			
≥30	0.76	0.52–1.11	0.93	0.69–1.25
				
**cause of trauma**				
traffic accident	reference			
non-traffic accident	1.28	0.85–1.92	1.66	1.22–2.24
Unknown	1.05	0.14–7.87	1.00	0.16–6.06
				
**GCS score on admission**				
mild (13-15)	reference			
moderate (9-12)	2.18	1.48–3.21	2.58	1.91–3.49
				
**Sex**				
Female	reference			
Male	1.78	1.14–2.78	1.09	0.79–1.52
				
**Age**				
<15	0.16	0.02–1.27	0.10	0.01–0.72
16-50	reference			
51-64	2.79	1.65–4.74	2.76	1.76–4.34
65-74	6.09	3.58–10.36	5.12	3.29–7.96
≥75	15.67	8.51–28.84	10.42	6.45–16.81
				
**dilatation of pupils**				
Unilateral	1.48	0.65–3.36	1.92	0.99–3.70
Bilateral	0.62	0.19–1.94	0.80	0.30–2.14
				
**hypotension (systolic BP < 90 mmHg)**	2.01	0.8–5.08	1.91	0.92–3.97
				
**bradycardia (HR < 60 beats per minute)**	1.26	0.56–2.85	0.95	0.49–1.84
				
**BT (≥37.5 °C)**	0.84	0.39–1.82	0.99	0.56–1.75
				
**ISS ≥ 16**	1.76	1.01–3.06	1.38	0.91–2.09
				
**hyperglycemia (BS ≥ 200 mg/dL)**	1.34	0.83–2.15	1.88	1.29–2.76
				
**hypoxia (PO_2_ < 60 mmHg)**	2.00	1.02–3.90	1.35	0.80–2.28
				
**hypercapnia (PCO_2_ > 45 mmHg)**	1.36	0.89–2.07	1.29	0.83–2.00
**hypocapnia (PCO_2_ < 35 mmHg)**	1.26	0.70–2.26	1.44	1.03–2.01
				
acidemia (pH < 7.3)	1.01	0.34–2.9	0.93	0.40–2.17
				
**TCDB classification (classification of TBI)**				
I	reference			
II	0.84	0.22–3.24	2.13	0.78–5.83
III	1.48	0.24–9.06	4.11	1.02–16.67
IV	0.92	0.07–11.99	2.57	0.34–19.33
V (evacuated)	0.51	0.14–1.83	1.21	0.47–3.13
VI (non-evacuated)	0.82	0.22–3.06	2.29	0.85–6.15
				
**operation**	0.81	0.45–1.45	0.62	0.41–0.92
				
**targeted temperature management (TTM)**	1.95	1.27–2.98	1.52	1.11–2.07
				
**skull fracture**	0.84	0.54–1.31	0.65	0.47–0.90
				
**intraventricular hemorrhage (IVH)**	2.57	1.06–6.28	3.72	1.85–7.46
				
**subarachnoid hemorrhage (SAH)**	2.19	1.45–3.31	2.26	1.66–3.07

**Table 3:** Age distribution of favorable and unfavorable outcomes at discharge stratified by sex.

Females		Total (N = 195)	Unfavorable (N = 89, 45.6%)	Favorable (N = 106, 54.4%)	*p*-Value
age	median, IQR	68 (41.0–79.0)	76 (68.0–82.5)	53 (20.8–69.3)	<0.001
age group					<0.001
<15	n, %	14 (7.2)	0 (0.0)	14 (13.2)	
16–50	n, %	40 (20.5)	6 (6.7)	34 (32.1)	
51–64	n, %	29 (14.9)	9 (10.1)	20 (18.9)	
65–74	n, %	46 (23.6)	28 (31.5)	18 (17.0)	
≥75	n, %	66 (33.9)	46 (51.7)	20 (18.9)	
					
**Males**		**Total (N = 521)**	**Unfavorable (N = 250, 47.9%)**	**Favorable (N = 272, 52.1%)**	
age	median, IQR	58.5 (35.0–71.0)	68 (56.8–78.0)	43.5 (25.0–62.0)	<0.001
age group					<0.001
<15	n, %	20 (3.8)	1 (0.4)	19 (7.0)	
16–50	n, %	186 (35.6)	48 (19.2)	138 (50.7)	
51–64	n, %	111 (21.3)	56 (22.4)	55 (20.2)	
65–74	n, %	113 (21.7)	70 (28.0)	43 (15.8)	
≥75	n, %	92 (17.6)	75 (30.0)	17 (6.3)	

## Data Availability

The datasets analyzed in this study are available from the Japan Neurotrauma Data Bank upon reasonable request.

## Supplementary Materials

The following supporting information can be downloaded at: https://www.mdpi.com/article/10.3390/jcm15052034/s1. Supplementary Table S1: Sex differences associated with unfavorable outcomes at 6 months in multivariable analysis; Supplementary Table S2: Transition matrix of 5-point GOS from discharge to 6 months (n = 521); Supplementary Table S3: Transition matrix of dichotomized outcome from discharge to 6 months (n = 521); Supplementary Table S4: Changes in 5-point GOS between discharge and 6 months according to sex; Supplementary Table S5: Changes in dichotomized outcome between discharge and 6 months according to sex.

## Abbreviations

The following abbreviations are used in this manuscript:

AIS	Abbreviated injury score
BS	blood sugar
BP	blood pressure
CI	confidence interval
CPA	cardiopulmonary arrest
CHF	congestive heart failure
CKD	chronic kidney disease
DM	diabetes mellitus
GCS	Glasgow Coma Scale
GOS	Glasgow Outcome Scale
HR	heart rate
HT	hypertension
ICP	intracranial pressure
IL-10	Interleukin-10
IQR	interquartile range
ISS	Injury Severity Score
IVH	intraventricular hemorrhage
JNTDB	Japan Neurotrauma Data Bank
OR	odds ratio
PO_2_	partial pressure of oxygen
PCO_2_	partial pressure of carbon dioxide
SAH	subarachnoid hemorrhage
SD	severe disability
MD	moderate disability
GR	good recovery
VS	vegetative state
TCDB	Traumatic Coma Data Bank Classification
TBI	traumatic brain injury
TTM	targeted temperature management
