# Supplementary material for: Sex and Age Differences in Outcomes of Traumatic Brain Injury: Findings from the Japan Neurotrauma Data Bank
Source: J Clin Med. 2026 Mar 6;15(5):2034. doi: 10.3390/jcm15052034 (PMC12986317; doi:10.3390/jcm15052034)
Supplement: Supplementary file 1 [file jcm-15-02034-s001.zip › jcm-4138012-supplementary.pdf]

Supplementary Table S1

Multivariable logistic regression analysis of factors associated with unfavorable functional outcomes at 6 months.

Adjusted odds ratios (ORs) with 95% confidence intervals (CIs) were calculated using multivariable logistic regression models. Male sex was used as the reference category.

Variable	Adjusted OR	95% CI	Crude OR	95% CI
Female (reference)				
Male	1.71	0.98–3.00	1.10	0.74–1.65

Supplementary Table S2

Transition matrix of 5-point Glasgow Outcome Scale (GOS) scores from hospital discharge to 6 months (n=521).

Rows represent 6-month GOS scores and columns represent discharge GOS scores. Bowker's test: $p < 0.001$; Wilcoxon signed-rank test: $p < 0.001$.

6 months ↓ / Discharge →	D	VS	SD	MD	GR	Total
D	128	3	2	1	0	134
VS	23	6	0	0	0	29
SD	2	49	5	1	0	57
MD	0	29	47	3	0	79
GR	0	10	54	158	0	222
Total	128	28	96	107	162	521

Supplementary Table S3

Transition matrix of dichotomized Glasgow Outcome Scale outcomes (favorable vs. unfavorable) from hospital discharge to 6 months (n=521).

Favorable outcome was defined as GOS 4–5 and unfavorable outcome as GOS 1–3. Bowker's test: $p < 0.001$; Wilcoxon signed-rank test: $p < 0.001$.

6 months ↓ / Discharge →	Unfavorable	Favorable	Total
Unfavorable	213	7	220
Favorable	39	262	301
Total	252	269	521

Supplementary Table S4

Changes in 5-point Glasgow Outcome Scale (GOS) scores between hospital discharge and 6 months according to sex.

The p-value was calculated using the chi-square test.

Change	Total n (%)	Male n (%)	Female n (%)	p-value
Improved	95 (18.2)	71 (18.8)	24 (16.8)	0.74
Worsened	21 (4.0)	14 (3.7)	7 (4.9)	
Unchanged	405 (77.7)	293 (77.5)	112 (78.3)	
Total	521 (100)	378 (72.6)	143 (27.4)	

Supplementary Table S5

Changes in dichotomized Glasgow Outcome Scale outcomes (favorable vs. unfavorable) between hospital discharge and 6 months according to sex.

The p-value was calculated using the chi-square test.

Change	Total n (%)	Male n (%)	Female n (%)	p-value
Improved	39 (7.5)	27 (7.1)	12 (8.4)	0.57
Worsened	7 (1.3)	4 (1.1)	3 (2.1)	
Unchanged	475 (91.2)	347 (91.8)	128 (89.5)	
Total	521 (100)	378 (72.6)	143 (27.4)	
